# Ultra-processed food consumption and associations with biomarkers of nutrition and inflammation in pregnancy: The Norwegian Environmental Biobank

**DOI:** 10.3389/fnut.2022.1052001

**Published:** 2022-12-08

**Authors:** Pieta Tasnim Kelsey, Eleni Papadopoulou, Tiril Cecilie Borge, Cecilie Dahl, Anne Lise Brantsæter, Iris Erlund, Helle Margrete Meltzer, Line Småstuen Haug, Ida Henriette Caspersen

**Affiliations:** ^1^Division of Climate and Environmental Health, Department of Food Safety, Norwegian Institute of Public Health, Oslo, Norway; ^2^Department of Community Medicine and Global Health, Institute of Health and Society, University of Oslo, Oslo, Norway; ^3^Global Health Cluster, Division for Health Services, Norwegian Institute of Public Health, Oslo, Norway; ^4^Cluster for Reviews and Health Technology Assessments, Division for Health Services, Norwegian Institute of Public Health, Oslo, Norway; ^5^Department of Government Services, Finnish Institute for Health and Welfare, Helsinki, Finland; ^6^Centre for Fertility and Health, Norwegian Institute of Public Health, Oslo, Norway

**Keywords:** pregnancy, C-reactive protein, ferritin, carotenoids, vitamins, essential elements, the Norwegian Mother, Father and Child Cohort Study

## Abstract

**Background:**

A high consumption of ultra-processed foods (UPFs) is often associated with low nutritional quality, but data on associations with biomarkers are scarce. We aimed to explore associations between UPF intake, diet quality, and concentrations of biomarkers of nutrition and inflammation measured in mid-pregnancy.

**Methods:**

This cross-sectional study included *n* = 2,984 pregnant women recruited during 2002–2008 in the Norwegian Mother, Father, and Child Cohort Study (MoBa). Concentrations of C-reactive protein (CRP) and 21 nutritional biomarkers including carotenes (α-carotene, β-carotene, γ-carotene, α-cryptoxanthin, β-cryptoxanthin, lutein, lycopene), vitamins [α-tocopherol, γ-tocopherol, 25-hydroxyvitamin D (25-OH-D), retinol], creatinine, elements (K, Na, Co, Cu, Mn, Mo, Se, Zn), and ferritin (Fe) were measured in blood and urine collected in mid-pregnancy. Habitual diet in pregnancy was assessed using a validated semi-quantitative food frequency questionnaire. We calculated the relative (%) energy contribution of UPF to overall intake according to the NOVA classification. We also applied a diet quality index (DQI) adapted to assess adherence to Norwegian dietary guidelines (DQI; min–max: 0–110, higher score meaning higher adherence). We present summary statistics for biomarker concentrations and explored associations between UPF intake or the DQI and measured biomarkers using adjusted linear, logistic, and generalized additive regression models.

**Results:**

Ultra-processed food intake was positively associated with biomarker concentrations of vitamin E (γ-tocopherol), creatinine, K, and Na [βs: 5.6 to 17% increase in biomarker concentration per interquartile range (IQR) increase in UPF intake] and negatively associated with carotenoids (α-carotene, β-carotene, γ-carotene, α-cryptoxanthin, β-cryptoxanthin, lutein, lycopene), vitamin A, Mo, and Se (βs: −2.1 to −18%). Inversely, high diet quality (i.e., the DQI) was positively associated with concentrations of carotenoids, vitamins [vitamin A (retinol) and D (25-OH-D)], and Se (β: 1.5 to 25%) and negatively associated with vitamin E (γ-tocopherol), creatinine, and Na (β: −4.8 to −8.3%). A weak, positive association was found between UPF and CRP (β: 5.4%, 95% CI 0.12–11%).

**Conclusion:**

High UPF intake was associated with reduced concentrations of nutrition biomarkers in mid-pregnancy. Associations in the opposite direction were found with high adherence to the Norwegian dietary guidelines, suggesting that the two dietary scoring systems capture diet quality in a mirrored manner in this population.

## Introduction

Poor nutritional status in pregnancy is associated with adverse health outcomes in the mother and child (1). In recent years, attention has been drawn to the consumption of ultra-processed foods (UPF). UPF can be defined as industrial formulations of foods whose major ingredients have undergone a series of physical, chemical, and biological processes (2). A high intake of UPF can result in poor nutritional status and excessive intake of salt, fats, and sugars, in addition to modified food substances, additives, and unwanted by-products or contaminants from extensive processing methods. UPF intake has been associated with obesity and development of non-communicable diseases, such as hypertension, diabetes, and dyslipidemia (3–7). However, whether UPF may affect health outcomes beyond its reduced nutritional value is not clear. While studies have shown how UPF intake relates to overall diet quality (8, 9), also in pregnancy (10, 11), data are scarce on the associations with biomarkers. Several *a priori* defined dietary indices have been developed to assess overall diet quality, also in pregnancy (12, 13). For assessment of intake according to the degree of food processing, the NOVA (not an acronym) classification is usually applied. Using NOVA, one classifies food groups according to the nature, extent, and purpose of industrial processing (2). The NOVA classification system is often used for studying associations between UPF intake and health outcomes (5), but usually not evaluated against nutritional biomarkers. Biomarkers in blood and urine are considered objective measures of nutrient status and widely used as a reference method in validation of dietary assessment methods (14). Few studies have, however, used biomarkers for evaluation of dietary quality indices.

This study aimed to (1) describe the concentrations and correlations of 22 nutrition and inflammation biomarkers measured in maternal blood sampled in pregnancy; and (2) explore associations between UPF consumption, a diet quality index (DQI) developed to assess adherence to Norwegian dietary guidelines, and concentrations of biomarkers of nutrition and inflammation status measured in pregnancy.

## Materials and methods

### Study participants

Our study is based on the first part of the Norwegian Environmental Biobank (NEB) project, which is a sub-study of the Norwegian Mother, Father, and Child Cohort Study (MoBa). MoBa is a population-based pregnancy cohort study conducted by the Norwegian Institute of Public Health (NIPH). Participants were recruited from all over Norway from 1999 to 2008 (15). The women agreed to participation in 41% of the pregnancies. Biological samples were obtained from mothers, fathers, and children (16). The cohort currently includes 114,500 children, 95,200 mothers, and 75,200 fathers. MoBa uses data from the medical birth registry (MBRN), which is a national health registry containing information about all births in Norway. The current cross-sectional study is based on version 12 of the quality-assured data files released for research in 2019.

**FIGURE 1:**
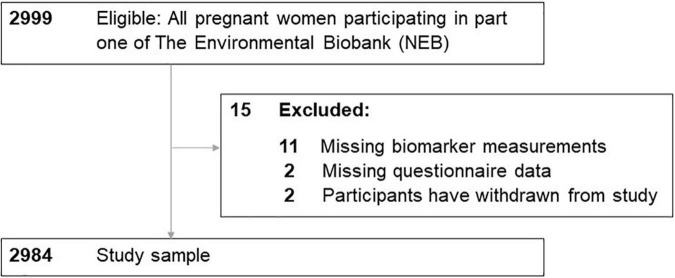
Inclusion of participants in the study sample. The Norwegian Environmental Biobank is a sub-study of the Norwegian Mother, Father, and Child Cohort Study (MoBa), including women who were pregnant in 2002–2008.

In a subsample of MoBa, a range of biomarkers have been analyzed in blood and urine samples with the purpose of biomonitoring nutrients and environmental contaminants for pregnant women (17) (part one of the NEB). Selection of pregnant women in MoBa into NEB was based on availability of biological samples from mid-pregnancy and at birth. Only women with live-born singletons, and who had answered all questionnaires up until 3 years after giving birth were eligible. The study sample is restricted to participants recruited from 2002 to 2008 because the MoBa food frequency questionnaire (FFQ) was included in the data collection from March 2002. Also, women with children with autism or suspected autism were excluded. A total of 2,999 pregnant women were included in NEB. For this study, we excluded *n* = 15 subjects (5%), of which *n* = 13 had missing data and *n* = 2 had withdrawn. Figure 1 outlines the flow of subjects for inclusion from the NEB sample.

### Assessment of dietary intake and construction of food indices

In MoBa, a semi-quantitative and validated FFQ was used to assess maternal dietary habits and intake of foods, beverages, and dietary supplements during the first half of pregnancy (18, 19). Intake in grams per day for 255 foods and beverages, assuming standard portion sizes, and energy and nutrient intakes were calculated using FoodCalc (20) and the Norwegian food composition database (21).

Using the NOVA classification system for grouping foods according to the degree of industrial processing (2), we categorized foods and beverages from the MoBa FFQ into four groups (1 = minimally processed, 2 = culinary ingredients, 3 = processed foods, and 4 = UPFs), Supplementary Table 1. All food items from the FFQ were classified into one of the NOVA groups by two nutritionists with detailed knowledge about the MoBa FFQ and Norwegian diet (22). By combining the NOVA grouping with estimated intakes based on the FFQ, we calculated the energy contribution by each NOVA group to the total daily intake, resulting in four scores (scale 0–100%) for each participant. From this point and throughout the manuscript we are using the term “UPF intake” or “energy contribution of UPF on total intake” to describe the relative contribution of NOVA group 4 to total intake.

A pregnancy DQI was constructed specifically for use in MoBa based on the FFQ and the Norwegian food-based dietary guidelines. Borge et al. describes the detailed methods and formula used (23). The DQI is based on the healthy eating index (HEI), a well-known tool for measuring food consumption patterns and diet qualities to provide healthy nutritional recommendations for the USA population (13). The DQI includes 13 components, where each component has a maximum score of 10 (apart from total fish and fatty fish, which each has a maximum score of 5), for a total score of 110. The DQI score (scale 0–110) reflects the adherence to the Norwegian dietary guidelines and higher score units indicate better diet quality.

### Assessment of biomarkers

The list of biomarkers measured in this study include CRP and ferritin measured in plasma, as well as nutrition biomarkers, including essential elements measured in whole blood (Cu, Mn, Mo, Se, Zn, in μg/L); vitamins including retinol (vitamin A, in mg/L), carotenoids (α-carotene, β-carotene, γ-carotene, α-cryptoxanthin, β-cryptoxanthin, lutein, lycopene, in mg/L), 25-hydroxy-vitamin-D (25-OH-D, in nmol/L), and tocopherols (α- and γ-tocopherol, in mg/L), measured in plasma; and Na, K and creatinine measured in urine (mmol/L).

During routine ultrasound visits around gestational week 18, MoBa women donated blood and urine samples (16). The biospecimen used in this study was collected from women who were in gestational week 18.5 (mean value, SD 1.3). Biochemical analyses of the nutritional and health related biomarkers were performed at the Department of Government Services (Biomarkers team), Finnish Institute for Health and Welfare (THL) in Helsinki, Finland. The laboratory (No. T077) has been accredited by the Finnish Accreditation Service (FINAS) and it fulfills the requirement of the standard SFS-EN ISO/IEC 17025:2017. Plasma CRP, ferritin, and vitamin D, as well as urinary K and Na, were measured using the Architect 8200ci integrated analyzer and assays developed for the purpose (Abbott Laboratories, Abbott Park, IL, USA). CRP (accredited method) was measured by the Multigent CRP Vario (CRPVa) assay, which is suitable for measuring CRP at variable assay ranges, including the low range requiring high sensitivity. P-Fe was analyzed by a chemiluminescent microparticle immune-assay (CMIA, ARCHITECT Ferritin assay, Abbott Laboratories). The Architect 25-(OH)-D assay (accredited method) was used for the determination of plasma vitamin D. The method is a high through-put automated chemiluminescent microparticle immunoassay, measuring both 25-(OH)-D2 and 25-(OH)-D3. Urinary K and Na were determined by Integrated Chip Technology (ICT) with ion-selective electrodes utilizing membranes selective to the ions (Abbott Laboratories). The laboratory participated in external quality assessment schemes organized by Labquality (Finland) and DEQAS (UK) for the above analytes.

Fat-soluble vitamins including retinol (vitamin A), carotenoids, and tocopherols (vitamin E) were also analyzed at the THL in Helsinki (Finland). High-performance liquid chromatography (HPLC) using an Agilent HPLC 1260 system with a diode array detector (Agilent Technologies Inc., Santa Clara, CA, USA) was utilized. Plasma samples were protected from light during extraction and chromatographic analysis. Extraction was performed by using ethanol, potassium chloride, ascorbic acid, hexane, and butylated hydroxytoluene. Carotenoids were detected at 450 nm, except β-lycopene which was detected at 472 nm. Tocopherols were detected at 292 nm and retinol at 326 nm. Peak height/internal standard ratios were compared to those of reference plasma, the values of which were traceable to NIST certified serum standards, 968e (National Institute of Standardization and Technology, Gaithersburg, MD, USA). Echinenone, tocol, and retinyl acetate were used as an internal standard. The element analysis in whole blood were performed at Department of Occupational and Environmental Medicine at Lund University, Sweden (17). All determinations (Co, Cu, Mn, Mo, Se, Zn) were performed with inductive coupled plasma mass spectrometry (ICP-MS; iCAP Q, Thermo Fisher Scientific, Bremen, GmbH) equipped with collision cell with kinetic energy discrimination and helium as collision gas.

### Other variables

The Medical Birth Registry of Norway and MoBa questionnaires provided lifestyle and sociodemographic factors. The following baseline characteristics were included: the women’s age at delivery (years), parity (primiparous/multiparous), pre-pregnancy body mass index (BMI) (kg/m^2^), completed educational level (low: ≤ 12 years, medium: 13–16 years; high: ≥ 17 years), maternal alcohol consumption during early pregnancy (yes/no), and smoking prior to or during pregnancy (no/sometimes/daily).

### Ethics

The establishment and data collection in MoBa was previously based on a license from the Norwegian Data Protection Authority and approval from the Regional Committee for Medical Research Ethics, and it is now based on regulations according to the Norwegian Health Registry Act. The current study was approved by the Regional Committee for Medical Research Ethics (ref. 2014/314).

### Statistical analysis

Associations between diet scores and nutritional biomarkers were explored using multiple linear regression models. We present the relative (%) increase in median biomarker concentrations and corresponding *p*-value of the associations in a volcano plot, which enables comparisons of the direction and magnitude of associations between all biomarkers and the two dietary indices. The outcome variables (biomarkers) were ln-transformed to approach normality. The relative (percent) change in concentrations of the biomarkers associated with a unit (c) change in the diet score was calculated by (exp(c*β)−1)*100% and corresponding confidence intervals by (exp(c*β ± z1−α/2*SE(β))−1)*100% with α = 0.05 and estimated βs and standard errors (SE) from the multiple regression analysis. We defined the unit c as the interquartile range (IQR, between first and third quartiles) to consider the observed range of the diet scores. The following covariates were considered as potential confounders and included as covariates: Age at delivery, pre-pregnancy BMI, education level, parity, alcohol consumption, and smoking during pregnancy. The variables with the largest proportion of missing data were pre-pregnancy BMI (3.6%) and early pregnancy alcohol consumption (10.8%). All adjusted analyses were performed based on complete case analysis. The linearity of associations between diet scores and nutritional biomarkers were inspected using non-parametric generalized additive models, using a restricted cubic spline with five knots as smoother. Bivariate correlations between dietary indices and biomarkers were investigated by Spearman’s rank correlation coefficient. As an additional analysis, we investigated associations between inflammatory markers CRP and ferritin and all NOVA groups 1–4 (minimally processed to ultra-processed), as well as the DQI, using logistic and linear regression models. Statistical analyses were performed in R, version 4.1.0.

## Results

The mean (SD) age of the study participants was 30 (4.2) years, and 51% were pregnant with their first child (Table 1). Most of the women had attained 13–16 years of education (45%), had a pre-pregnancy BMI between 18.5–24.9 kg/m^2^ (63%), had no reported alcohol consumption in early pregnancy (87%), and were non-smokers (92%). Mean energy contributions for each NOVA group were 29% from foods in group 1 (minimally processed), 3% from group 2 (culinary ingredients), 22% from group 3 (processed), and 46% from group 4 (ultra-processed). Mid-pregnancy concentrations of biomarkers in plasma, urine and whole blood are shown in Table 2.

**TABLE 1:** Study sample characteristics (*n* = 2,984).

	Study sample, (*n* = 2,984)
**Age (years), mean (SD)**	30 (4.2)
**Parity, *n* (%)**	
Nulliparous	1,529 (51)
Multiparous	1,455 (49)
**Education, *n* (%)**	
≤ 12 years	737 (25)
13–16 years	1,345 (45)
≥ 17 years	717 (24)
Missing	63 (2)
**Pre-pregnancy BMI, *n* (%)**	
< 18.5 kg/m^2^	82 (3)
18.5–24.9 kg/m^2^	1,897 (63)
25.0–29.9 kg/m^2^	719 (24)
≥ 30.0 kg/m^2^	231 (8)
Missing	55 (2)
**Alcohol in pregnancy, *n* (%)**	
No	2,594 (87)
Yes	67 (2)
Missing	323 (11)
**Smoking during pregnancy, *n* (%)**	
No	2,741 (92)
Occasionally	68 (2)
Daily	114 (4)
Missing	61 (2)
**NOVA classification, relative (%) energy contribution to overall intake,^1^ mean (SD)**	
Minimally processed foods (group 1)	29 (8.7)
Processed culinary ingredients (group 2)	3 (3.3)
Processed foods (group 3)	22 (10.7)
Ultra-processed foods (group 4)	46 (14.1)
**Diet quality index (DQI), mean (SD)^1,2^**	83 (9.0)

Numbers are mean (SD) or *n* (%).

^1^NOVA classification and DQI was defined for *n* = 2,797 subjects with available FFQ data.

^2^Possible scoring range was 0–110, observed range was 46–104. Higher score indicates better quality.

**TABLE 2:** Summary statistics of biomarker concentrations measured in mid-pregnancy blood and urine samples (*n* = 2,984).

	Unit	Matrix	*n*	Mean	*SD*	Min.	P25	Median	P75	Max.
**Carotenoids**										
α-Carotene	mg/L	Plasma	2,984	0.07	0.05	0.004	0.038	0.06	0.09	0.46
β-Carotene	mg/L	Plasma	2,984	0.29	0.15	0.030	0.185	0.256	0.36	1.7
γ-Carotene	mg/L	Plasma	2,984	0.05	0.01	0.01	0.03	0.045	0.054	0.12
α-Cryptoxanthin	mg/L	Plasma	2,984	0.03	0.01	0.009	0.02	0.03	0.04	0.08
β-Cryptoxanthin	mg/L	Plasma	2,984	0.12	0.07	0.02	0.07	0.010	0.15	0.79
Lycopene	mg/L	Plasma	2,984	0.4	0.15	0.03	0.3	0.4	0.5	1.1
Lutein	mg/L	Plasma	2,984	0.19	0.06	0.05	0.15	0.18	0.22	0.46
**Tocopherols (vitamin E)**										
α-Tocopherol	mg/L	Plasma	2,984	13	2.5	5.7	11	13	15	31
γ-Tocopherol	mg/L	Plasma	2,984	0.91	0.39	0.17	0.63	0.83	1.1	3.02
Vitamin D (25-OH-D)^1^	nmol/L	Plasma	2,981	51	19	13	38	50	63	182
Vitamin A (retinol)	mg/L	Plasma	2,984	0.44	0.08	0.16	0.38	0.43	0.48	0.81
Creatinine	mmol/L	Urine	2,984	7.6	5.1	0.50	3.5	6.7	10	35
**Elements**										
K	mmol/L	Urine	2,984	50	30	4.0	27	45	69	204
Na	mmol/L	Urine	2,975	142	57	21	96	137	182	352
Co^2^	μg/L	Whole Blood	2,968	0.19	0.19	0.03	0.09	0.15	0.23	3.09
Cu^2^	μg/L	Whole Blood	2,968	1,554	250	610	1,388	1,542	1,702	3,633
Mn^2^	μg/L	Whole Blood	2,968	10	03.8	2.4	8.2	10	12	53
Mo^2^	μg/L	Whole Blood	2,968	0.73	0.44	0.14	0.49	0.63	0.82	7.2
Se^2^	μg/L	Whole Blood	2,968	105	23	42	89	102	117	353
Zn^2^	μg/L	Whole Blood	2,968	4,824	933	1,153	4,240	4,805	5,366	11,057
**Inflammation markers**										
C-Reactive protein (CRP)	mg/L	Plasma	2,982	6.4	7.7	0.11	2.6	4.6	7.6	189
Ferritin (Fe)^2^	μg/L	Plasma	2,984	43	34	3.2	20	33	55	304

^1^25-hydroxyvitamin D.

^2^Summary statistics for element concentrations in this study sample are previously published in Caspersen et al. (17).

Ultra-processed food intake was negatively, but weakly correlated with the DQI (rho = −0.3, Supplementary Figure 1). While negatively correlated on a group level, the individual correlation varied; 2.2% of the women were in the first quartile (Q1) for both UPF and DQI, and 1.2% were in Q4 for both scores. In comparison, 28% were in completely opposite quartiles (Q1 and Q4) for the two indices, with 14% in each direction, respectively. Across quartiles of the DQI (Q1–Q4), the median values of UPF intake were 57% (Q1), 50% (Q2), 44% (Q3), and 35% (Q4). Biomarker concentrations were also correlated (Supplementary Figure 1).

**FIGURE 2:**
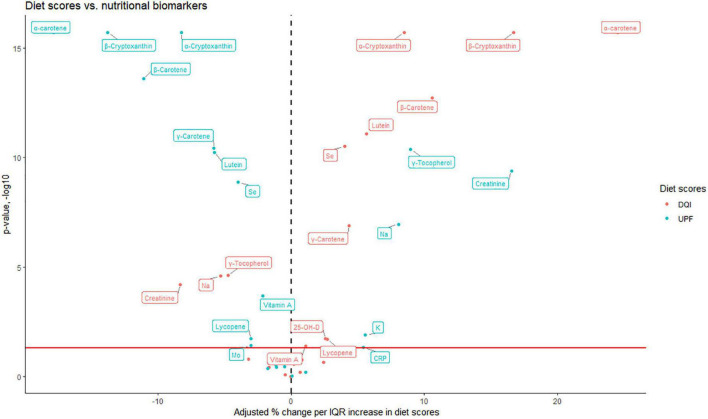
Adjusted relative (%) change in biomarker concentration according to an interquartile range (IQR, 25th–75th percentile) increase in diet scores (DQI and UPF), estimated by linear regression. Red horizontal line is indicating the –log10 transformed value corresponding to *p*-value = 0.05, and an increasing value on the *y*-axis corresponds to a decrease in the *p*-value. Only biomarkers with *p*-value < 0.05 are indicated with a text box. Adjusted for age at delivery, parity, pre-pregnancy BMI, education level, alcohol intake, and smoking status. CRP, C-reactive protein; DQI, diet quality index; UPF, ultra-processed food; 25-OH-D, 25-hydroxyvitamin D.

**FIGURE 3:**
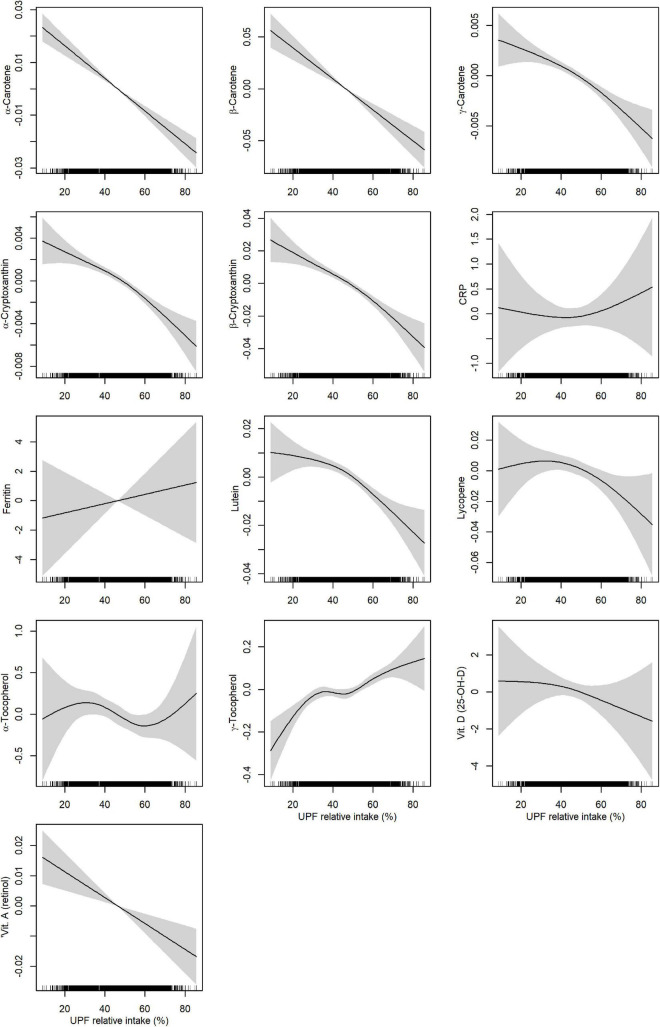
Adjusted associations between relative intake of ultra-processed food (UPF) and biomarkers measured in plasma. Associations were estimated by non-parametric generalized additive models using a restricted cubic spline with five knots as smoother. Adjusted for age at delivery, parity, pre-pregnancy BMI, education level, alcohol intake, and smoking status. CRP, C-reactive protein.

**FIGURE 4:**
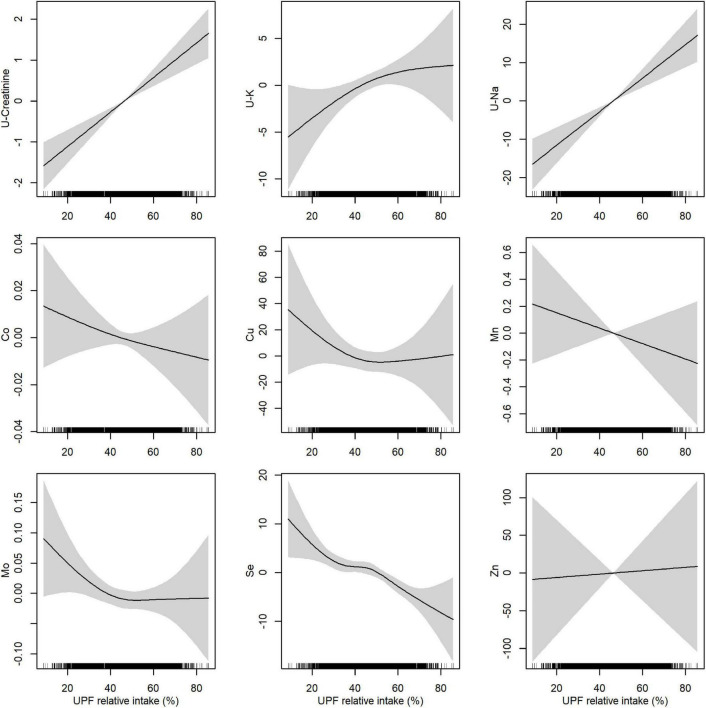
Adjusted associations between relative intake of ultra-processed food (UPF) and biomarkers measured in urine (indicated by “U-”) and whole blood. Associations were estimated by non-parametric generalized additive models using a restricted cubic spline with five knots as smoother. Adjusted for age at delivery, parity, pre-pregnancy BMI, education level, alcohol intake, and smoking status.

To examine how biomarker concentrations were associated with UPF intake, we plotted the magnitude and significance of linear associations for the relative intake of UPF together with associations with the DQI in a volcano plot (Figure 2). An IQR increase (from the 25th to the 75th percentile) of energy contribution from UPF was positively associated with vitamin E (γ-tocopherol), creatinine, K, and Na (β: 5.6 to 16.5%; CI lower: 1.2 to 11.1%; CI upper: 10.1 to 22.2%), and negatively associated with all carotenoids (α-carotene, β-carotene, γ-carotene, α-cryptoxanthin, β-cryptoxanthin, lutein, lutein, lycopene), vitamin A, Mo, and Se (β: −2.1 to −17.9%; CI lower: −3.2 to −20.9%; CI upper: −1.0 to −14.6%). The DQI was positively associated with all carotenoids (α-carotene, β-carotene, γ-carotene, α-cryptoxanthin, β-cryptoxanthin, lutein, lycopene), vitamins (vitamin A and D), and Se (β:1.5 to 24.5%; CI lower: 0.05 to 20%; CI upper: 2.1 to 28.8%), and negatively associated with vitamin E (γ-tocopherol), creatinine, and Na (β: −4.8 to −8.3%; CI lower: −6.9 to −12.1%; CI upper:−2.6 to −4.3%). All unadjusted and adjusted effect estimates, and corresponding *p*-values are shown in Supplementary Table 2. Non-parametric generalized additive models showed clear linear relationships between UPF intake and concentrations of carotenoids, vitamin A (Figure 3), creatinine and Na (Figure 4).

Additional analysis showed weak, but positive associations between high UPF intake and inflammation biomarkers CRP and ferritin (Supplementary Tables 3–5). For instance, an IQR increase in UPF intake was associated with a 5.4% (CI lower: 0.12%, CI upper: 11%) increase in CRP concentration. No linear association was found between UPF intake and ferritin (Supplementary Table 3). However, the odds of high ferritin concentration (> 70 vs. 15–69 μg/L) was increased (odds ratio 1.4, CI lower: 1.1, CI upper: 1.9) when comparing the upper vs. lower quartile of UPF intake (Supplementary Tables 4, 5).

## Discussion

In this cross-sectional study based on a large cohort of Norwegian women, we estimated associations between biomarker concentrations and (1) UPF intake (according to the NOVA classification), and (2) scores from a DQI assessing adherence to Norwegian dietary guidelines. Our findings indicated that high consumption of UPFs was positively associated with urinary Na concentration, reflecting high intake of salt, and negatively associated with nutritional biomarkers, including carotenoids, vitamin A, selenium, and molybdenum, reflecting lower intake of vegetables, wholegrain and other foods that are recommended in a healthy diet. The associations between UPF consumption and the DQI with measured biomarkers showed inverse patterns, suggesting that the two dietary scoring systems capture high and low diet quality in a mirrored manner in this population. These findings are in line with those in a recent study where UPF intake was associated with lower diet quality assessed by HEI during pregnancy and postpartum in a USA study sample (10).

While concentrations of most vitamins were positively associated with DQI and negatively associated with the relative contribution from UPF, the opposite association was seen for γ-tocopherol. A likely explanation is that tocopherols are commonly used as antioxidants to inhibit peroxidation of fats and lipids in foods and thus is associated with the intake of UPFs (24). Notably, the biomarker concentrations reported in this study were measured in different matrices (whole blood, plasma, urine). The concentrations reported should be interpreted in light of the many physiological changes during pregnancy, such as hemodilution (25).

Our findings indicated weak, but positive associations between high intake of UPF and plasma CRP and ferritin concentrations, which are both markers of inflammation (26, 27). Increased inflammation is associated with pregnancy complications, and may therefore be of concern (28, 29). CRP has been shown to increase during pregnancy, but the patterns of change are inconsistent (30), and the specific effect of diet quality or dietary components on inflammation remains unclear. However, a recent systematic review described significant associations between dietary patterns and inflammatory markers during pregnancy (31). While diets rich in fruits, vegetables and whole-grain have been found to decrease CRP levels, meat-based western diets were associated with increased CRP levels in non-pregnant adults (32, 33). A suggested mechanism for the latter association is that UPF consumption could affect inflammatory status through changes in the gut microbiota (34). Moreover, a high degree of UPFs in the diet often leads to excess caloric intake and weight gain in comparison to an unprocessed diet (35), thereby indirectly increasing inflammation. These potential intermediate factors were not explored in this study.

The main strength of our study is the use of a validated FFQ and availability of a wide range of nutrition and inflammation biomarkers in a large sample of pregnant women. Additionally, access to extensive information about the participating women from the MoBa questionnaires allowed us to investigate associations between dietary indices and biomarkers while adjusting for a range of possible confounders.

There are some limitations to our study. MoBa participants are more often supplement users, non-smokers, primiparous, and have higher education compared to the general Norwegian pregnant population (36). Still, when comparing diet quality and biomarkers of nutrients within the same individuals, we expect that self-selection into the cohort is less problematic than in studies of exposure-disease outcomes. One can speculate that reported associations with dietary indices and biomarker status found in this sample could be of even greater magnitude in a more heterogeneous population (in terms of variation in exposure and biomarker concentrations) than studied here. Although we were able to adjust for a range of potential confounders, there is always a possibility that our results may be biased by unmeasured confounders, such as lifestyle, genetics and other physiological or dietary factors. We did not examine supplement use in this study. Associations between the two dietary indices and biomarkers may differ between users and non-users of supplements, and between users of supplements with different content. A previous study based on the same study sample showed that use of multimineral supplements was associated with increased concentrations of Mo and Se (17). However, if supplement use correlates to the DQI and UPF intake in a similar manner, we would still expect that the DQI and UPF intake are associated with biomarker concentrations in opposite directions.

We defined UPF consumption by applying the NOVA framework to consumption data of food groups assessed using an FFQ which was not designed for this specific purpose. This may limit the comparability with other studies. Also, this study is based on data collected during 2002–2008, and the content of the foods classified as UPF in this study may not be directly comparable to foods consumed now. The MoBa FFQ did not include sufficient detail for exact categorization into NOVA groups. Some groups may be incorrect as they include items that may or may not include added sugar, and products that may or may not be homemade and in that case less processed. Although the interpretability of the NOVA classification system is discussed (37, 38), it is extensively used as a processing-based classification system in studies of diet quality and health outcomes, which facilitates comparisons between studies. Another limitation is that we have not described the intake of nutrients by UPF intake. However, the contrast and exclusive categorization of women by the DQI and UPF is still supporting that high UPF is related to poor nutritional status. Also the DQI was developed relying on the investigator’s choice on how to create the index, specifically with regards to the number and weighting of components while calculating the DQI score (23). It has been argued that the accuracy of dietary indices depending on *a priori* approach can be limited by lack of dietary knowledge in terms of diet-health relationship and uncertain methodologies during index construction (39). However, the FFQ used in this study was specifically developed to assess habitual diet in the target population and provides reasonably valid dietary intake estimates (18). Additionally, utilizing composite measures of overall diet quality is considered less prone to misreporting compared to estimations of single nutrient intake (18, 39).

In conclusion, we found that a high relative contribution of UPF to total energy intake coincided with reduced concentrations of nutrition biomarkers in pregnancy. Inverse associations were found for high adherence to the Norwegian food-based dietary guidelines. The opposite directions of the associations with biomarkers seen for UPF intake and the DQI suggest that the two dietary scoring systems capture high and low diet quality in a mirrored manner in this population. Our findings shed light on the effect of UPF intake on nutritional status, using nutritional biomarkers, which should be considered when studying associations between consumption of UPFs in pregnancy and maternal and child health outcomes.

## Data availability statement

The original contributions presented in the study are included in the article/Supplementary material, further inquiries can be directed to the corresponding author/s.

## Ethics statement

The studies involving human participants were reviewed and approved by the Regional Committee for Medical Research Ethics (ref. 2014/314). The patients/participants provided their written informed consent to participate in this study.

## Author contributions

PTK, EP, TCB, ALB, and IHC prepared the data. PTK, EP, and IHC performed the statistical analyses. PTK and IHC wrote the first draft of the manuscript. EP, TCB, ALB, IE, HMM, LSH, CD, and IHC interpreted the results and revised the manuscript. IE was responsible for supervising the laboratory analyses. All authors read and approved the final version.
