# Supplementary material for: Ultra-processed food consumption and associations with biomarkers of nutrition and inflammation in pregnancy: The Norwegian Environmental Biobank
Source: Front Nutr. 2022 Dec 8;9:1052001. doi: 10.3389/fnut.2022.1052001 (PMC9772468; doi:10.3389/fnut.2022.1052001)
Supplement: Supplementary file 1 [file Data_Sheet_1.docx]

Supplementary Material

**Kelsey PT, et al. Ultra-processed food consumption and associations with biomarkers of nutrition and inflammation in pregnancy: The Norwegian Environmental Biobank.**

**Table S1.** Classification of foods and beverages in the MoBa FFQ according to the NOVA classification.

| \| **Group 1 -unprocessed or minimally processed (grouping based on 114 items in FFQ)** \| **Group 2 –**  **culinary ingredients (grouping based on 16 items in FFQ)** \| **Group 3 –**  **processed foods (grouping based on 48 items in FFQ)** \| **Group 4 –**  **ultra-processed foods (grouping based on 77 items in FFQ)** \| \| --- \| --- \| --- \| --- \| \| Coffee \| Butter \| Beer and wine \| Alcoholic beverages \| \| Egg \| Dressing \| Bread, wholemeal \| Bacon \| \| Fish \| Honey \| Caviar \| Biscuits \| \| Fruits \| Oil \| Cereals, mixed \| Bread, white \| \| Meat, cooked \| Probiotic milk \| Cheese \| Cereals with added sugars \| \| Milk and cream \| Soy/rice milk \| Cod liver \| Drinks with artificial sweeteners \| \| Nuts \| Sugar \| Crisp bread, musli bread \| Fish sticks \| \| Oatmeal \|  \| Fish spreads \| French fries \| \| Liver \|  \| Fish, smoked or canned \| Hamburgers \| \| Orange juice \|  \| Meat, fried \| Ice creams \| \| Seafood \|  \| Minced meat, oven-baked gratins, stews \| Lunch meats \| \| Sorbet \|  \| Pancakes, waffles \| Margarines \| \| Syrup \|  \| Peanutbutter \| Meat spreads \| \| Tea \|  \| Sardines in oil \| Pastries \| \| Vegetables \|  \| Yoghurt, flavoured \| Pizza \| \| Yoghurt, no flavor \|  \|  \| Powdered soups and sauces \| \|  \|  \|  \| Sausages \| \|  \|  \|  \| Savoury snacks \| \|  \|  \|  \| Store bought condiments \| \|  \|  \|  \| Sugar added to foods/beverages \| \|  \|  \|  \| Sugary spreads \| \|  \|  \|  \| Sweets/candies \| |
| --- | --- | --- | --- | --- | --- | --- | --- | --- | --- | --- | --- | --- | --- | --- | --- | --- | --- | --- | --- | --- | --- | --- | --- | --- | --- | --- | --- | --- | --- | --- | --- | --- | --- | --- | --- | --- | --- | --- | --- | --- | --- | --- | --- | --- | --- | --- | --- | --- | --- | --- | --- | --- | --- | --- | --- | --- | --- | --- | --- | --- | --- | --- | --- | --- | --- | --- | --- | --- | --- | --- | --- | --- | --- | --- | --- | --- | --- | --- | --- | --- | --- | --- | --- | --- | --- | --- | --- | --- | --- | --- | --- | --- |

**Table S2.** Relative change (%) in nutrition biomarker concentration associated with interquartile range (IQR) increase in diet quality index (DQI) and ultra-processed food (UPF) intake, estimated by linear regression

|  | **DQI** | | | | **UPF** | | | |
| --- | --- | --- | --- | --- | --- | --- | --- | --- |
|  | Relative % change  (95% CI),  unadjusted | p-value | Relative % change  (95% CI),  adjusted^1^ | p-value | Relative % change  (95% CI),  unadjusted | p-value | Relative % change  (95% CI),  adjusted^1^ | p-value |
| α-Carotene | 33.4 (29.2, 37.8) | <0.001 | 24.5 (20.4, 28.8) | <0.001 | -24.4 (-27.0, -21.6) | <0.001 | -17.9 (-21.0, -14.6) | <0.001 |
| β-Carotene | 15.6 (12.8, 18.5) | <0.001 | 10.6 (7.7, 13.6) | <0.001 | -15.3 (-17.6, -12.9) | <0.001 | -11.1 (-13.7, -8.4) | <0.001 |
| γ-Carotene | 6.3 (4.8, 7.8) | <0.001 | 4.3 (2.7, 6.0) | <0.001 | -7.6 (-9.0, -6.1) | <0.001 | -5.8 (-7.4, -4.1) | <0.001 |
| α-Cryptoxanthin | 12.2 (10.4, 14.1) | <0.001 | 8.5 (6.6, 10.4) | <0.001 | -11.8 (-13.4, -10.2) | <0.001 | -8.3 (-10.0, -6.4) | <0.001 |
| β-Cryptoxanthin | 21.7 (18.7, 24.9) | <0.001 | 16.7 (13.6, 20.0) | <0.001 | -18.3 (-20.6, -15.9) | <0.001 | -13.8 (-16.4, -11.0) | <0.001 |
| Lutein | 9.3 (7.7, 10.9) | <0.001 | 5.6 (4.0, 7.3) | <0.001 | -9.1 (-10.6, -7.6) | <0.001 | -5.8 (-7.4, -4.1) | <0.001 |
| Lycopene | 3.7 (1.6, 5.8) | <0.001 | 2.7 (0.4, 5.1) | 0.019 | -3.9 (-6.1, -1.7) | <0.001 | -3.0 (-5.4, -0.5) | 0.018 |
| α -Tocopherol | 0.5 (-0.5, 1.4) | 0.344 | 0.1 (-1.0, 1.1) | 0.903 | -1.6 (-2.6, -0.5) | 0.003 | -0.9 (-2.0, 0.3) | 0.143 |
| γ -Tocopherol | -8.2 (-10.1, -6.3) | <0.001 | -4.8 (-6.9, -2.6) | <0.001 | 13.2 (10.6, 15.8) | <0.001 | 9.0 (6.2, 11.8) | <0.001 |
| Vitamin A (retinol) | 1.5 (0.5, 2.4), | 0.002 | 1.1 (0.0, 2.1) | 0.040 | -2.5 (-3.5, -1.6) | <0.001 | -2.1 (-3.2, -1.0) | <0.001 |
| Vitamin D (25-OH-D) | 4.3 (2.3, 6.3) | <0.001 | 2.6 (0.4, 4.8) | 0.019 | -3.4 (-5.5, -1.3) | 0.001 | -1.1 (-3.4, 1.3) | 0.372 |
| Creatinine | -11.5 (-14.8, -8.1) | <0.001 | -8.3 (-12.1, -4.3) | <0.001 | 20.0 (15.1, 25.2) | <0.001 | 16.5 (11.1, 22.2) | <0.001 |
| K | -3.0 (-6.2, 0.3) | 0.075 | -1.7 (-5.3, 2.1) | 0.379 | 7.2 (3.2, 11.2) | <0.001 | 5.6 (1.2, 10.1) | 0.013 |
| Na | -7.0 (-9.1, -4.9) | <0.001 | -5.3 (-7.7, -2.9) | <0.001 | 10.9 (8.1, 13.7) | <0.001 | 8.1 (5.0, 11.2) | <0.001 |
| Co | 2.7 (-0.8, 6.3) | 0.132 | 2.4 (-1.4, 6.4) | 0.217 | -0.9 (-4.6, 3.0) | 0.642 | -1.8 (-5.9, 2.5) | 0.417 |
| Cu | -0.1 (-0.9, 0.7) | 0.822 | 0.2 (-0.7, 1.1) | 0.275 | 0.2 (-0.7, 1.1) | 0.671 | -0.5 (-1.5, 0.5) | 0.343 |
| Mn | 0.0 (-1.7, 1.7) | 0.992 | 0.0 (-1.9, 1.9) | 0.962 | -1.0 (-2.9, 0.8) | 0.277 | -1.1 (-3.2, 1.0) | 0.286 |
| Mo | 2.0 (-0.3, 4.3) | 0.095 | 0.7 (-1.9, 3.3) | 0.620 | -4.5 (-6.9, -2.1) | <0.001 | -3.0 (-5.8, -0.2) | 0.038 |
| Se | 5.1 (4.0, 6.2) | <0.001 | 4.0 (2.8, 5.2) | <0.001 | -5.8 (-6.9, -4.7) | <0.001 | -4.0 (-5.2, -2.7) | <0.001 |
| Zn | 0.4 (-0.6, 1.4) | 0.410 | 0.8 (-0.3, 1.9) | 0.172 | 0.3 (-0.8, 1.4) | 0.614 | 0.0 (-1.2, 1.3) | 0.943 |

^1^Adjusted for age, BMI, education, alcohol consumption, parity and smoking.

**Table S3.** Crude and adjusted^1^ associations between relative % change in inflammation markers and interquartile range (25^th^-75^th^ percentile) increase in DQI and NOVA scores, estimated by linear regression (n=2984)

|  | CRP^*^ | | Ferritin^*^ | |
| --- | --- | --- | --- | --- |
|  | Unadjusted, % (95 % CI) | Adjusted^*^ % (95 % CI) | Unadjusted, % (95 % CI) | Adjusted^*^ % (95 % CI) |
| DQI *(Score 0-110)* | **-10 (-14, -6.1)** | -3.2 (-7.5, 1.3) | -0.72 ( -4.3, 3.0) | -0.47 (-4.4, 3.6) |
| NOVA 1^2^ | **-7.7 (-11, -3.8)** | -2.7 (-6.7, 1.4) | 0.31 (-3.1, 3.8) | 0.31 (-3.1, 3.8) |
| NOVA 2^2^ | **-5.5 (-7.9, -2.9)** | **-2.7 (-5.2, -0.06)** | 1.1 (-1.1, 3.4) | 1.0 (-1.3, 3.4) |
| NOVA 3^2^ | **-5.3 (-10, -0.47)** | -2.2 (-7.1, 2.9) | 0.48 (-3.7, 4.8) | -0.7 (-5.0, 3.9) |
| NOVA 4^2^ | **13 (7.9, 19)** | **5.4 (0.12, 11)**^3^ | -1.1 (-5.1, 3.1) | 1.1 (-3.4, 5.7)**^4^** |

^1^Adjusted for age, BMI, education, alcohol consumption, parity and smoking.

^2^ Group 1: minimally processed; group 2, culinary ingredients, group 3, processed; group 4, ultra-processed foods. Unit of measurement is the relative (%) contribution to overall energy intake from the given food group.

^3^ p-value=0.045

^4^ p-value=0.64

**Table S4.** Adjusted^1^ odds ratios and 95% confidence intervals (ORs, 95% CI) for inflammation biomarkers (CRP and Ferritin) according to DQI and NOVA groups 1-4 in quartiles, estimated by multiple logistic regression (n=2984)

|  | **CRP (6-10 vs. <5 mg/L), adjusted OR (95% CI)** | **CRP (>10 vs. <5 mg/L), adjusted OR (95% CI)** | **Ferritin (<15 vs. 15-69 µg/L), adjusted OR (95% CI)** | **Ferritin (>70 vs. 15-69 µg/L), adjusted OR (95% CI)** |
| --- | --- | --- | --- | --- |
| *DQI (Score 0-110)* |  |  |  |  |
| Quartile 1 | Ref. (1) | Ref. (1) | Ref. (1) | Ref. (1) |
| Quartile 2 | 0.9 (0.7, 1.2) | 0.9 (0.6, 1.2) | 1.1 (0.8, 1.5) | 1.0 (0.8, 1.3) |
| Quartile 3 | 0.8 (0.6, 1.1) | 0.7 (0.5, 1.0) | 0.8 (0.6, 1.1) | 0.8 (0.6, 1.1) |
| Quartile 4 | 0.9 (0.7, 1.2) | **0.6 (0.4, 0.8)** | 0.9 (0.7, 1.3) | 0.8 (0.6, 1.0) |
| *NOVA 1 Minimally processed foods ^2^* |  |  |  |  |
| Quartile 1 | Ref. (1) | Ref. (1) | Ref. (1) | Ref. (1) |
| Quartile 2 | 0.8 (0.6, 1.0) | 0.7 (0.5, 1.0) | 1.0 (0.7, 1.4) | **0.7 (0.5, 0.9)** |
| Quartile 3 | 0.8 (0.6, 1.0) | 0.8 (0.6, 1.1) | 1.0 (0.7, 1.4) | 0.9 (0.7, 1.1) |
| Quartile 4 | 0.8 (0.6, 1.0) | 0.7 (0.5, 1.0) | 0.9 (0.7, 1.3) | **0.7 (0.5, 0.9)** |
| *NOVA 2 Culinary ingredients ^2^* |  |  |  |  |
| Quartile 1 | Ref. (1) | Ref. (1) | Ref. (1) | Ref. (1) |
| Quartile 2 | 0.9 (0.7, 1.2) | 0.8 (0.5, 1.0) | 0.7 (0.5, 1.0) | 0.8 (0.6, 1.0) |
| Quartile 3 | 0.8 (0.6, 1.0) | 0.7 (0.5, 1.0) | 0.7 (0.5, 1.0) | 0.7 (0.6, 1.0) |
| Quartile 4 | **0.6 (0.5, 0.8)** | **0.4 (0.3, 0.6)** | 0.8 (0.6, 1.1) | **0.6 (0.4, 0.7)** |
| *NOVA 3 Processed foods ^2^* |  |  |  |  |
| Quartile 1 | Ref. (1) | Ref. (1) | Ref. (1) | Ref. (1) |
| Quartile 2 | 1.1 (0.8, 1.4) | 0.9 (0.7, 1.2) | 0.7 (0.5, 1.0) | 1.1 (0.8, 1.4) |
| Quartile 3 | 0.9 (0.7, 1.2) | 0.8 (0.6, 1.1) | 0.7 (0.5, 1.0) | 0.9 (0.7, 1.2) |
| Quartile 4 | 1.0 (0.8, 1.3) | 0.8 (0.6, 1.1) | 0.8 (0.6, 1.1) | 1.0 (0.7, 1.3) |
| *NOVA 4 Ultraprocessed foods ^2^* |  |  |  |  |
| Quartile 1 | Ref. (1) | Ref. (1) | Ref. (1) | Ref. (1) |
| Quartile 2 | 1.1 (0.8, 1.4) | 1.0 (0.7, 1.4) | 0.9 (0.6, 1.2) | 1.1 (0.9, 1.5) |
| Quartile 3 | 1.0 (0.8, 1.3) | 1.0 (0.7, 1.5) | 1.1 (0.8, 1.5) | 1.1 (0.8, 1.5) |
| Quartile 4 | 1.3 (1.0, 1.7) | **1.7 (1.2, 2.4)** | 1.1 (0.8, 1.5) | **1.4 (1.1, 1.9)** |

^1^Adjusted for age, parity, pre-pregnancy BMI, education, alcohol consumption and smoking

^2^ Group 1: minimally processed; group 2, culinary ingredients, group 3, processed; group 4, ultra-processed foods. Unit of measurement is the relative (%) contribution to overall energy intake from the given food group.

**Table S5.** Unadjusted odds ratios and 95% confidence intervals (ORs, 95% CI) for association between inflammation status (CRP and Ferritin) and dietary indices DQI and NOVA groups 1-4, in quartiles, estimated by logistic regression (n=2984)

|  | **CRP (6-10 vs. <5 mg/L), unadjusted OR (95% CI)** | **CRP (>10 vs. <5 mg/L), unadjusted OR (95% CI)** | **Ferritin (<15 vs. 15-69 µg/L), unadjusted OR (95% CI)** | **Ferritin (>70 vs. 15-69 µg/L), unadjusted OR (95% CI)** |
| --- | --- | --- | --- | --- |
| *DQI (Score 0-110)* |  |  |  |  |
| Quartile 1 | Ref. (1) | Ref. (1) | Ref. (1) | Ref. (1) |
| Quartile 2 | 0.9 (0.7, 1.2) | 0.8 (0.6, 1.1) | 0.9 (0.7, 1.2) | 0.8 (0.6, 1.1) |
| Quartile 3 | 0.8 (0.6, 1.0) | **0.7 (0.5, 0.9)** | 0.8 (0.6, 1.0) | **0.7 (0.5, 0.9)** |
| Quartile 4 | 0.9 (0.7, 1.1) | **0.5 (0.4, 0.7)** | 0.9 (0.7, 1.1) | **0.5 (0.4, 0.7)** |
| *NOVA 1 Minimally processed foods ^2^* |  |  |  |  |
| Quartile 1 | Ref. (1) | Ref. (1) | Ref. (1) | Ref. (1) |
| Quartile 2 | 0.8 (0.6, 1.0) | 0.7 (0.5, 0.9) | 0.9 (0.7, 1.2) | 0.7 (0.5, 0.9) |
| Quartile 3 | 0.8 (0.7, 1.1) | 0.7 (0.6, 1.0) | 1.1 (0.8, 1.5) | 0.8 (0.6, 1.1) |
| Quartile 4 | 0.8 (0.6, 1.0) | 0.6 (0.4, 0.8) | 0.9 (0.7, 1.3) | 0.6 (0.5, 0.8) |
| *NOVA 2 Culinary ingredients ^2^* |  |  |  |  |
| Quartile 1 | Ref. (1) | Ref. (1) | Ref. (1) | Ref. (1) |
| Quartile 2 | 1.0 (0.7, 1.2) | 0.8 (0.6, 1.1) | 0.7 (0.5, 0.9) | 0.9 (0.7, 1.2) |
| Quartile 3 | 0.9 (0.7, 1.1) | 0.8 (0.6, 1.1) | 0.7 (0.5, 1.0) | 0.8 (0.6, 1.1) |
| Quartile 4 | 0.7 (0.6, 0.9) | 0.5 (0.4, 0.7) | 0.8 (0.6, 1.1) | 0.7 (0.5, 0.9) |
| *NOVA 3 Processed foods ^2^* |  |  |  |  |
| Quartile 1 | Ref. (1) | Ref. (1) | Ref. (1) | Ref. (1) |
| Quartile 2 | 1.1 (0.8, 1.4) | 0.9 (0.7, 1.3) | 0.9 (0.7, 1.3) | 1.1 (0.8, 1.4) |
| Quartile 3 | 0.9 (0.7, 1.2) | 0.8 (0.6, 1.2) | 0.8 (0.6, 1.1) | 0.9 (0.7, 1.1) |
| Quartile 4 | 1 (0.8, 1.3) | 0.8 (0.6, 1.1) | 1.0 (0.7, 1.3) | 0.9 (0.7, 1.2) |
| *NOVA 4 Ultraprocessed foods ^2^* |  |  |  |  |
| Quartile 1 | Ref. (1) | Ref. (1) | Ref. (1) | Ref. (1) |
| Quartile 2 | 1.1 (0.8, 1.4) | 1 (0.7, 1.4) | 0.9 (0.6, 1.2) | 1.1 (0.9, 1.5) |
| Quartile 3 | 1 (0.8, 1.3) | 1 (0.7, 1.5) | 1.0 (0.7, 1.3) | 1.1 (0.8, 1.4) |
| Quartile 4 | 1.3 (1, 1.7) | **1.7 (1.2, 2.4)** | 1.0 (0.8, 1.4) | **1.5 (1.1, 1.9)** |


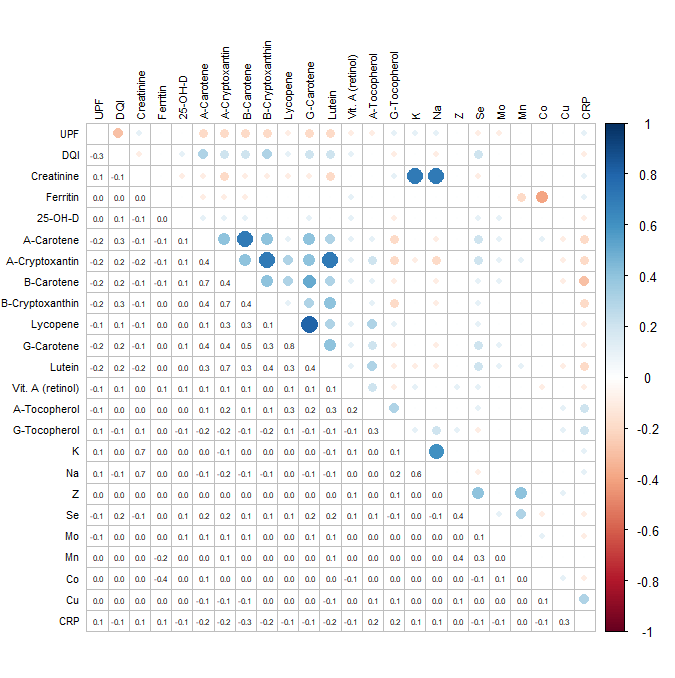


**Supplementary Figure S1.** Spearman correlations between dietary scores (UPF, DQI) and biomarkers measured in pregnancy. Increased circle size depicts stronger correlation. UPF, ultra-processed food (relative (%) intake, defined by NOVA classification system); DQI, diet quality index, UPF; CRP, C-reactive protein.
